# Development and Validation of Telephone Cognitive Testing for Community-Dwelling Older Adults (TCTCOA) in China

**DOI:** 10.3390/bs15030384

**Published:** 2025-03-19

**Authors:** Jiming Guo, Xiaodan Xue, Asad Ur Rehman Awan, Ying Wang, Tianyong Chen

**Affiliations:** 1 Institute of Psychology, Chinese Academy of Sciences, Beijing 100101, China; guojiming3@163.com (J.G.); xuexd@psych.ac.cn (X.X.); asadrehman9400@gmail.com (A.U.R.A.); xlyx_2022@163.com (Y.W.); 2 Department of Psychology, University of Chinese Academy of Sciences, Beijing 100049, China; 3 Chengdu Shishi Chengfei High School, Sichuan 610073, China

**Keywords:** cognitive function, telephone-based assessment, community-dwelling older adults

## Abstract

With the global acceleration of population ageing, cognitive health remains critical to the well-being of older adults. This study aimed to develop and validate Telephone Cognitive Testing for Community-dwelling Older Adults (TCTCOA), a culturally and contextually tailored cognitive assessment tool designed for healthy, community-dwelling older adults in China. TCTCOA included five cognitive domains—episodic memory, working memory, processing speed, executive function, and abstract reasoning and concept formation—assessed using culturally adapted tasks. A sample of 112 community-dwelling older adults aged 60 and above participated in the study. Sixty-eight participants completed TCTCOA via telephone and face-to-face modalities, alongside the Montreal Cognitive Assessment (MoCA) for validation. Pearson’s correlations, structural validity, and convergent validity were analyzed to evaluate the tool. TCTCOA demonstrated strong correlations between telephone and face-to-face modes (*r* = 0.72) and moderate correlations with the MoCA. Subtests showed no ceiling or floor effects, and the composite scores followed a normal distribution. The tool’s structural validity was supported by factor analysis, identifying general cognitive ability and efficiency as core components. TCTCOA is a valid, reliable, and accessible telephone-based cognitive assessment tool. It is suitable for healthy older adults in community settings, offering a practical alternative to traditional face-to-face cognitive evaluations. Its design overcomes cultural, educational, and logistical barriers, making it an effective resource for cognitive health monitoring in China.

## 1. Introduction

The global pace of population ageing is accelerating. According to the World Health Organization (WHO), by 2030, one in six people worldwide will be aged 60 years or older. China, representing a significant proportion of the global population, is experiencing substantial demographic ageing. This shift presents a critical societal challenge, particularly in relation to cognitive health conditions such as mild cognitive impairment (MCI) and dementia. Given the absence of effective treatments for these conditions, early detection and intervention are paramount. This highlights the urgent need for monitoring cognitive health in healthy elderly populations. Preventive strategies should primarily focus on this group, as they predominantly reside in communities. Along with the growing emphasis on health promotion (Haralambous et al., 2004; Fox, 2010) and proactive health initiatives (China GotPsRo, 2016; Liu et al., 2022), there is an increasing awareness among older adults in China about the importance of cognitive well-being. Many are actively seeking information about their cognitive status. However, despite this rising awareness, cognitive health assessments are not routinely incorporated into health examinations for the elderly in Chinese communities. This creates significant barriers to accessing the necessary evaluation resources. Therefore, there is a pressing need for an accessible, community-specific cognitive testing tool tailored for older adults in China. Such a tool would help bridge this gap and support efforts to monitor and promote cognitive health in this vulnerable population.

Compared to traditional face-to-face assessments, telephone-based tools offer greater accessibility for older adults. These tools can overcome limitations posed by factors such as transportation issues, physical disabilities, and technological constraints (e.g., the need for internet connectivity and smart devices for video conferencing or app-based assessments; (Marra et al., 2020)). As a result, they expand access to a broader segment of the elderly population. In recent years, particularly following the COVID-19 pandemic, telephone-based tools have gained traction. They serve as complementary alternatives to in-person assessments, offering contactless solutions that have demonstrated reliability (Castanho et al., 2014; Carlew et al., 2020). A review by Carlew et al. (2020) identified 43 existing telephone-based cognitive assessment tools. Most of these tools were designed primarily as screening instruments to differentiate between MCI, dementia, and normal cognitive status. However, these tools were not suitable for assessing healthy older adults, as they were often oversimplified and could fail to accurately capture the cognitive status of this population. Several tools have been developed for use with healthy older adults. The Brief Test of Adult Cognition by Telephone (BTACT) (Tun & Lachman, 2006) and the Cognitive Telephone Screening Instrument (COGTEL) (Kliegel et al., 2007) were among the most widely utilized. Both tools extended the cognitive domains typically covered by standard screening instruments. BTACT aligned with contemporary theories of cognitive aging (Tun & Lachman, 2006), and COGTEL aimed to provide a more comprehensive global assessment of cognitive function while avoiding ceiling effects (Kliegel et al., 2007). The cognitive domains assessed by BTACT included episodic verbal memory, working memory span, executive function, reasoning, and processing speed. The tasks used to evaluate these domains included immediate recall of a 15-word list, delayed recall of the same list, backward digit span, category fluency, and number series completion (Tun & Lachman, 2006). The cognitive domains assessed by COGTEL encompassed verbal short-term memory, verbal long-term memory, working memory, verbal fluency (executive functioning), inductive reasoning, and prospective memory. The tasks used for these assessments included a verbal paired-associate memory test (immediate recall), a delayed-retrieval test, a backward digit span test, letter fluency test, category fluency test for professions, number series completion task, and an event-based task (Kliegel et al., 2007).

BTACT and COGTEL were developed and implemented in the United States and Europe, respectively, where cultural differences compared to China must be taken into account. Additionally, the education level of the target populations for these existing tools does not align with that of older adults in China. The education level of this demographic group is notably lower compared to that of developed countries, with 86.1% of individuals aged 60 and older not having completed high school (Office of the Leading Group of the State Council for the Seventh National Population Census, 2021). In contrast, 87.8% of individuals aged 65 and above in the United States have attained at least a high school diploma (U.S. Census Bureau, 2024). Given these considerations, we developed a multi-domain cognitive assessment tool, Telephone Cognitive Testing for Community-dwelling Older Adults (TCTCOA), referring to contemporary cognitive ageing theories (Salthouse, 1994; Blazer et al., 2015; Harada et al., 2013; Anderson & Craik, 2017; Bryan & Luszcz, 2000; Park & Festini, 2017; Cadar et al., 2018) and existing tools such as BTACT and COGTEL. We selected five domains to comprehensively assess cognitive functioning in healthy Chinese older adults: episodic memory, working memory, processing speed, executive function, and abstract reasoning and concept formation. Each domain was evaluated through well-established tasks commonly used in cognitive assessments for healthy individuals. Considering cultural differences in language use, in the domain of episodic memory, we adapted the verbal paired associates subtest from the Wechsler Memory Scale-Revised (WMS-R) (Wechsler, 1987) into Chinese, ensuring the words were familiar to Chinese older adults. We selected animal category fluency to assess executive function, which is a semantic category that remains clear across languages and cultures (Ardila et al., 2006). Given the differences in education level, we did not include the number series completion task, as it is relatively difficult (LeFevre & Bisanz, 1986) and may result in floor effects among older adults (Quereshi & Smith, 1998). Instead, we used the verbal clock test (Cercy, 2012) as an alternative to assess the abstract reasoning and concept formation. This test was designed to minimize the influence of cultural and education background.

In summary, this study aimed to develop a multi-domain cognitive assessment tool tailored for elderly individuals in Chinese communities, enabling them to better understand their cognitive health status and take proactive steps to address ageing. The proposed tool, TCTCOA, was designed to differentiate itself from tools intended for MCI or dementia screening. It is free from ceiling and floor effects, adaptable to individuals with lower education levels, and easy to access.

## 2. Materials and Methods

### 2.1. Participants

This study included community-dwelling older adults aged 60 years and above. Participants were recruited from Beijing, China, between August and September 2023. A total of 112 older adults were initially enrolled. Exclusion criteria included a history of neurological or psychiatric disorders, as well as hearing impairments. The study was approved by the Ethics Committee of the Institute of Psychology, Chinese Academy of Sciences (IPCAS). All participants provided informed consent prior to participation.

### 2.2. Materials

Episodic memory was assessed using the verbal paired associates subtest adapted from the Wechsler Memory Scale-Revised (WMS-R) (Wechsler, 1987). The verbal paired associates consisted of twelve pairs of words, six relevant/easy pairs and six irrelevant/difficult pairs. The test requirements, examples, and the twelve word pairs were professionally recorded, and the words in the pairs were read at 1 s intervals, with 2 s intervals between pairs. The trials began with the playing of a recording, after which the tester showed the subjects the first word of each pair and asked for the word associated with it, with both the pairing and recall prompts presented in a different order. Easy word pairs were scored as 1 point, difficult word pairs were scored as 2 points, and the task score was the combined score of the two trials (0–36).

Working memory was assessed using the backward digit span test from the Wechsler Intelligence Scale-Revised (WAIS-R) (Wechsler, 1981). Subjects heard a series of numbers, two to eight numbers long, and were asked to repeat the number sequence in reverse order. Two trials were given for each sequence length, and if the first trial was passed, the subject went directly to the next sequence until both tests were incorrect. The first pass was scored as 2 points, the second pass was scored as 1 point, and the task score was a combined score (0–14).

Processing speed was assessed using the backward counting task from the BTACT (Tun & Lachman, 2006). Subjects were given 30 s to count backwards from 100 as quickly as possible. The task score was the total number of correctly reported digits, excluding errors and skipped digits.

Executive function was assessed using a category fluency task (Ardila et al., 2006). Subjects were asked to say as many words as possible in the category of animal within 60 s. The score was determined by the number of allowable words, excluding repetitions, while considering terms such as birds, fish, and other species as valid animal names, including dragons.

Abstract reasoning and concept formation were evaluated using the verbal clock test (Cercy, 2012). The test consisted of 10 questions, each presenting a scenario where the hour and minute hands were pointing to specific numbers. Subjects were required to verbally indicate the time without the aid of a clock, paper, or pen. Two points were awarded for correct responses on both the hour and minute, one point for a correct response on only one of the two components, and zero points for incorrect responses to both components, with a total possible score ranging from 0 to 20 across all 10 questions.

### 2.3. Procedure

All participants underwent cognitive assessment using TCTCOA. Of the 112 participants, 68 were first assessed by telephone, followed by a face-to-face assessment 2 weeks later. These participants also completed the Beijing version of the Montreal Cognitive Assessment (MoCA) during face-to-face assessment. The remaining 48 participants were assessed by telephone only. The telephone assessments were recorded using a mobile phone or computer to allow for later scoring. To ensure the validity of the assessments, participants were instructed not to use any external aids during the cognitive tests.

### 2.4. Data Analysis

All statistical analyses were performed using IBM SPSS Statistics, version 26.0. The distribution of scores in TCTCOA was assessed using the Kolmogorov–Smirnov test and descriptive statistics. To explore the effects of different measurement modes, Pearson’s correlation and *t*-tests were conducted. Bootstrap resampling was used to estimate the confidence intervals of key correlations. Structural validity was evaluated through factor analysis and the examination of the correlation matrix. Convergent validity was assessed by comparing the telephone results with the Montreal Cognitive Assessment (MoCA).

## 3. Result

### 3.1. Characteristics of the Participants

This study involved 112 older adults aged 61 to 83 years (*M* = 68.66 years, *SD* = 4.84 years), including 41 males and 71 females. The participants’ level of education ranged from 5.5 to 17 years (*M* = 11.13, *SD* = 2.41), and they were an essentially healthy sample on a 5-point scale of 1 (very good) to 5 (very bad) for self-rated health status (*M* = 2.61, *SD* = 0.75). Table 1 shows the composite scores of TCTCOA and the scores of the five subtests grouped by age, education, and self-rated health status. The composite score is the mean of the standardized z-scores of the five subtests. ANOVA showed significant differences in verbal paired associates, backward counting, and composite scores among older adults grouped by education level. Education had no significant effect on backward counting, verbal clock test, and category fluency. Self-assessed health status had a significant effect on verbal paired associates scores, as indicated by a *t*-test, *t*(110) = 2.12, *p* < 0.05.

### 3.2. Distribution of Scores in TCTCOA

The distribution of composite scores in TCTCOA was examined using the Kolmogorov–Smirnov test, and the composite scores followed a normal distribution (K-S *z* score = 0.057, *p* > 0.05) (*n* = 112). The mean scores, minimum and maximum values, score ranges, and 5th, 25th, 50th, 75th, and 95th percentiles for the five cognitive tests of TCTCOA are shown in Table 2. Scores on the five subtests ranged from 12 to 54. On the verbal paired associates, backward digit span, and verbal clock test, 75% of participants did not achieve a perfect score, with only about 5% achieving a perfect score on the verbal clock test.

### 3.3. The Effects of the TCTCOA Administration Modes

Table 3 shows the descriptive data and Pearson correlations between telephone administration and face-to-face administration for the five subtests of TCTCOA. The different test modes of the five subtests showed moderate to strong correlations. The correlation between the combined telephone and face-to-face test scores was *r* = 0.72 (*p* < 0.001), indicating a strong correlation. A *t*-test comparing telephone and face-to-face administration revealed no significant effect of test mode on backward digit span (*t*(67) = 0.60, *p* = 0.549) or category fluency (*t*(67) = 0.68, *p* = 0.499). However, significant differences were observed for verbal paired associates (*t*(67) = 5.01, *p* < 0.001), backward counting (*t*(67) = 2.72, *p* = 0.008), and verbal clock (*t*(67) = 5.02, *p* < 0.001). Mean scores for the face-to-face test were consistently higher than those for the telephone test.

### 3.4. Structural Validity of TCTCOA

TCTCOA was analyzed for structural validity (112-person sample data). As shown in Table 4, the Pearson correlations between each cognitive domain and the composite score of TCTCOA were high and positive, ranging from 0.51 to 0.65. In addition, we observed that the correlation of each domain with the composite score of TCTCOA was significantly higher than the correlation with another domain, which demonstrates the ability of cognitive domains to differentiate. These correlations indicate the validity of structural correlations.

We assessed the factor structure of TCTCOA by exploratory factor analysis, extracting principal component factors from the five subtest scores, and applying eigenvalues greater than 1 to determine the number of factors, using varimax orthogonal rotation. Two factors appeared in the factor analysis. The factor loadings for each subtest are shown in Table 5.

### 3.5. Convergent Validity of TCTCOA

Comparing the distributions of the MoCA and TCTCOA, the distribution of the composite score of TCTCOA for the 68 individuals obeyed a normal distribution (K-S *z* score = 0.092, *p* > 0.05), and the distribution of the MoCA scores for the 68 individuals was not normal (K-S *z* score = 0.120, *p* < 0.02) but negatively skewed.

The Spearman’s correlation between the composite score of TCTCOA for telephone administration and the total MoCA score was *r* = 0.44 (*p* < 0.001), 95% CI [0.216, 0.632]. The Spearman’s correlation between the composite score of the telephone cognitive assessment tool for face-to-face administration and the total MoCA score was *r* = 0.47 (*p* < 0.001), 95% CI [0.257, 0.637].

## 4. Discussion

TCTCOA offers a multi-domain, telephone-based evaluation of cognitive function for healthy older adults residing in Chinese communities. The test is not affected by ceiling effects, is easy to administer and score, and typically takes 15 min to complete.

Different administration modes do not largely influence the scores of TCTCOA. The correlation of the composite score of the two modes was relatively high (*r* = 0.72). The scores of five subtests also showed moderate to strong correlations across administrations. These findings are consistent with results from several previous studies (Carlew et al., 2020; Tun & Lachman, 2006; Kliegel et al., 2007). In some tasks, higher scores were observed in face-to-face administration, particularly in the verbal paired associates and backward counting tasks. These results align with prior research (Tun & Lachman, 2006) and are likely due to practice effects, as same materials were used across administrations, especially in the verbal paired associates task where two rounds of word-pair recall were included. A similar practice effect is also noted in the newly adapted verbal clock task, possibly due to better strategy use in the face-to-face test.

The composite score of the telephone administration was moderately correlated with the total MoCA scores, indicating convergent validity. The distribution of participants’ scores on the five subtests demonstrated a significant range of variability and did not exhibit a ceiling effect. The composite scores of the five cognitive tests followed a normal distribution, ensuring that TCTCOA is not limited by ceiling effects. In contrast, the MoCA scores exhibited negative skewness, indicating it is better suited for detecting cognitive impairment (Nasreddine et al., 2005), whereas TCTCOA is more appropriate for assessing cognitive function in community-dwelling older adults.

Factor analysis of TCTCOA revealed two distinct factors. Factor 1, which included episodic memory, working memory, and abstract reasoning and concept formation, represents general cognitive ability. Factor 2, which included category fluency and processing speed, reflects cognitive efficiency. Processing speed is one of the strongest predictors of verbal fluency performance (Shao et al., 2014; Amunts et al., 2020). In tests involving time pressure, tasks significantly influenced by processing speed reflect cognitive efficiency. These findings are similar to those of the Wechsler Intelligence Test, which uses two index scores: the General Ability Index and the Cognitive Proficiency Index (Weiss, 2010).

TCTCOA is a practical tool for cognitive assessment, particularly in settings where face-to-face testing is not feasible. However, there are several limitations in the current study that should be addressed in future research. First, our study primarily focused on the development of TCTCOA, including the selection of cognitive domains and corresponding tasks. The sample size, however, was relatively small and unevenly distributed across demographic variables, limiting its representativeness. Future studies should prioritize larger, more representative samples, with the goal of establishing national norms. The generalizability of the findings would be enhanced by increasing the sample size and employing stratified sampling based on factors such as age, education level, region, and socio-economic status. Second, while our study focused on the agreement between different test modes, reliability was assessed by comparing telephone and face-to-face testing. Future research could incorporate test–retest reliability measures to assess the tool’s stability over time. Additionally, a parallel version of TCTCOA could be created to evaluate parallel-test reliability and further explore any practice effects observed in the study. Third, although TCTCOA primarily targets healthy ageing, its clinical validity requires further examination. Future studies should assess its sensitivity and validate its applicability by testing it on individuals in the early stages of neurodegeneration.

Despite these limitations, telephone-based cognitive assessment remains valuable, especially in low- and middle-income countries or regions where in-person testing may not be feasible. TCTCOA offers a reliable and straightforward method for evaluating cognitive function in these contexts. It also provides an appropriate way to address the needs and initiatives of proactive health. In addition to traditional cognitive scores, TCTCOA provides insights into speech characteristics. Tasks such as word memory, fluency tasks, and backward counting provide speech-induced topics (Garcia et al., 2020). Tests are conducted, and audio can be recorded in a quiet environment and analyzed by automatic speech recognition (ASR). ASR can be used to score and analyze speech responses, examining features such as silence duration and repetition, which can be predictive of a later AD diagnosis (Robin et al., 2021; Gosztolya et al., 2019).

## 5. Conclusions

In summary, cognitive abilities are essential for the health and independence of individuals, particularly older adults. TCTCOA offers a convenient alternative to face-to-face testing for assessing cognitive functioning in the Chinese elderly population.

## Figures and Tables

**Table 1:** Descriptive data on participants’ composite scores and subtest scores on Telephone Cognitive Testing for Community-dwelling Older Adults (TCTCOA) (*n* = 112).

Variable	*n*	Composite Score	Verbal Paired Associates	Backward Digit Span	Category Fluency	Backward Counting	Verbal Clock Test
Age (years)							
60–69	65	0.04 ± 0.60	13.05 ± 6.28	7.51 ± 2.87	19.92 ± 5.35	31.80 ± 7.96	15.15 ± 3.27
70+	47	−0.05 ± 0.57	12.13 ± 5.64	7.04 ± 2.63	19.19 ± 4.86	32.57 ± 9.91	15.00 ± 2.95
		*t* = 0.76	*t* = 0.80	*t* = 0.88	*t* = 0.74	*t* = −0.46	*t* = 0.26
Education							
Primary/junior high school	29	−0.31 ± 0.48	9.00 ± 3.84	6.24 ± 2.08	18.17 ± 5.71	31.07 ± 7.25	14.66 ± 3.73
High school/technical secondary school	50	0.02 ± 0.60	14.4 ± 6.37	7.42 ± 2.88	19.36 ± 4.27	30.94 ± 9.46	14.96 ± 2.9
Junior college/undergraduate	33	0.24 ± 0.54	13.24 ± 5.75	8.09 ± 2.91	21.27 ± 5.52	34.85 ± 8.64	15.67 ± 2.88
		*F* = 7.58 ***	*F* = 8.69 ***	*F* = 3.68 *	*F* = 3.02	*F* = 2.30	*F* = 0.89
Health							
Good	39	0.11 ± 0.60	14.28 ± 6.13	7.82 ± 3.25	19.56 ± 5.95	32.72 ± 7.86	15.23 ± 2.62
Fair/bad	73	−0.06 ± 0.57	11.79 ± 5.8	7.04 ± 2.46	19.64 ± 4.70	31.81 ± 9.3	15.01 ± 3.38
		*t* = 1.48	*t* = 2.12 *	*t* = 1.31	*t* = −0.08	*t* = 0.52	*t* = 0.35

* *p* < 0.05, *** *p* < 0.001.

**Table 2:** Distribution of scores on the five subtest scores of Telephone Cognitive Testing for Community-dwelling Older Adults (TCTCOA) (*n* = 112).

	*M* ± *SD*	Rang	Min	Max	Percentiles				
					5	25	50	75	95
Verbal paired associates	12.66 ± 6.01	30	0	30	4.65	8.00	12.00	16.75	26.00
Backward digit span	7.31 ± 2.77	12	2	14	4.00	5.00	7.00	9.00	13.00
Category fluency	19.62 ± 5.14	28	4	32	10.00	17.00	19.50	23.00	29.00
Backward counting	32.13 ± 8.80	54	13	67	19.30	27.00	31.00	37.00	47.35
Verbal clock test	15.09 ± 3.12	15	5	20	9.00	13.00	16.00	17.00	20.00

**Table 3:** Scores in five subtests of Telephone Cognitive Testing for Community-dwelling Older Adults (TCTCOA) across administration modes (*n* = 68).

Subscale	Telephone	Face-to-Face	*r*	*p*
	*M* ± *SD*	*M* ± *SD*		
Episodic memory (verbal paired associates)	11.31 ± 5.16	14.50 ± 5.71	0.54	<0.001
Working memory (backward digit span)	6.37 ± 2.29	6.53 ± 2.03	0.48	<0.001
Verbal fluency (category fluency)	19.13 ± 5.40	19.56 ± 5.46	0.55	<0.001
Processing speed (backward counting)	32.60 ± 9.74	34.82 ± 9.94	0.77	<0.001
Abstract reasoning and concept formation (verbal clock test)	14.88 ± 3.51	16.63 ± 2.95	0.62	<0.001

**Table 4:** Correlation between cognitive domains of Telephone Cognitive Testing for Community-dwelling Older Adults (TCTCOA) and with composite scores (*n* = 112).

Variable	1	2	3	4	5	6
1. Composite score	1.00					
2. Episodic memory	0.65 ***	1.00				
3. Working memory	0.61 ***	0.43 ***	1.00			
4. Verbal fluency	0.51 ***	0.06	0.05	1.00		
5. Processing speed	0.56 ***	0.15	0.08	0.25 **	1.00	
6. Abstract reasoning and concept formation	0.60 ***	0.25 **	0.23 *	0.12	0.17	1.00

* *p* < 0.05, ** *p* < 0.01, *** *p* < 0.001.

**Table 5:** Factor loadings of Telephone Cognitive Testing for Community-dwelling Older Adults (TCTCOA) (*n* = 112).

	1	2
Episodic memory	0.80	
Working memory	0.81	
Abstract reasoning and concept formation	0.54	
Verbal fluency		0.79
Processing speed		0.76

## Data Availability

The data presented in this study are available on request from the first author.
